# False detection of rifampicin resistance using Xpert^®^ MTB/RIF Ultra assay due to an A451V mutation in *Mycobacterium tuberculosis*

**DOI:** 10.1093/jacamr/dlab101

**Published:** 2021-08-11

**Authors:** Margaret M Fitzgibbon, Emma Roycroft, Gerard Sheehan, Anne-Marie Mc Laughlin, Keith Ian Quintyne, Elaine Brabazon, Mary O’Meara, Peter R Flanagan, A -Louise Seagar, Ian F Laurenson, Joseph Keane, Thomas R Rogers

**Affiliations:** Irish Mycobacteria Reference Laboratory, St James’s Hospital, Dublin, Ireland; Department of Clinical Microbiology, Trinity College Dublin, Ireland; Irish Mycobacteria Reference Laboratory, St James’s Hospital, Dublin, Ireland; Department of Clinical Microbiology, Trinity College Dublin, Ireland; Mater Misericordiae University Hospital, Dublin, Ireland; School of Medicine, University College Dublin, Ireland; Department of Respiratory Medicine, St James’s Hospital, Dublin, Ireland; Clinical Medicine, Trinity College Dublin, Ireland; Department of Public Health North-East, Meath, Ireland; Department of Public Health North-East, Meath, Ireland; Department of Public Health East, Health Service Executive, Dublin, Ireland; Irish Mycobacteria Reference Laboratory, St James’s Hospital, Dublin, Ireland; Department of Clinical Microbiology, Trinity College Dublin, Ireland; Scottish Mycobacteria Reference Laboratory, Edinburgh, Scotland; Scottish Mycobacteria Reference Laboratory, Edinburgh, Scotland; Department of Respiratory Medicine, St James’s Hospital, Dublin, Ireland; Clinical Medicine, Trinity College Dublin, Ireland; Irish Mycobacteria Reference Laboratory, St James’s Hospital, Dublin, Ireland; Department of Clinical Microbiology, Trinity College Dublin, Ireland

## Abstract

**Background:**

In a 12 month period, three Irish-born adult cases with pulmonary TB were initially diagnosed by Xpert^®^ MTB/RIF Ultra assay, which detected a rifampicin resistance-conferring mutation prompting treatment as potential MDR cases.

**Methods:**

Further laboratory investigations on the cultured isolates included GenoType MTBDR*plus* assay, phenotypic drug susceptibility tests using the BD BACTEC MGIT culture system and MIC broth microdilution tests. Sequencing of the *rpoB* gene was performed using Sanger sequencing and WGS.

**Results:**

Phenotypic drug susceptibility tests determined the isolates to be rifampicin susceptible. Molecular investigations identified an A451V (codon 532) mutation in the *Mycobacterium tuberculosis rpoB* gene that has not previously been found to cause rifampicin resistance. Genome sequencing revealed that the three isolates’ genomes differed by ≤5 SNPs, indicating a high likelihood of recent transmission events. Furthermore, a cluster of six related *M. tuberculosis* isolates from our in-house typing database showed four were highly related; all were rifampicin susceptible and lacked this mutation.

**Conclusions:**

False detection of rifampicin resistance, albeit rare, should be considered possible with Xpert^®^ MTB/RIF Ultra assay, particularly in low TB incidence settings. Confirmatory sequencing methods should be performed to prevent the unnecessary use of second-line anti-tuberculous drugs.

## Introduction

Subsequent to its endorsement by WHO in 2010 the Xpert^®^ MTB/RIF assay, succeeded by the Xpert^®^ MTB/RIF Ultra (Cepheid, Sunnyvale, CA, USA), became an established test for rapid diagnosis of pulmonary TB and detection of mutations within the 81 bp *rpoB* gene rifampicin-resistance determining region (RRDR).^1^^,^^2^ When an RRDR mutation is detected using the Xpert^®^ MTB/RIF Ultra assay, WHO guidelines recommend starting treatment with an MDR-TB regimen.^3^ However, in cases where the *rpoB* mutation is not associated with phenotypic rifampicin resistance, this could result in overuse of second-line anti-tuberculous drugs .

We report three cases of pulmonary TB where the Xpert^®^ MTB/RIF Ultra assay result for rifampicin resistance mutation(s) led to initial therapy with an MDR-TB drug regimen. Subsequent confirmation of rifampicin susceptibility enabled a change in regimen to include rifampicin in two cases with successful completion of therapy. Treatment in the third case did not include rifampicin because of the patient’s underlying immunocompromise and lack of clarity at the time on the clinical relevance of the A451V mutation. Genome sequencing of a further six isolates associated with this cluster showed that four were closely related to the index isolates but lacked an A451V mutation. Further epidemiological investigations failed to identify a link between any of these cases .

## Materials and methods

Three sputum samples that were processed as part of routine diagnostic testing in the Irish Mycobacteria Reference Laboratory (IMRL) in a 12 month period between 2018 and 2019 were included in this study .

The Xpert^®^ MTB/RIF Ultra assay was performed as per manufacturer’s instructions (Cepheid). The Xpert^®^ MTB/RIF Ultra v2 assay was used for sample 1 whereas Xpert^®^ MTB/RIF Ultra v3 assay was used for samples 2 and 3.

All three sputum samples were cultured using the BACTEC MGIT 960 culture system according to the manufacturer’s instructions (Becton Dickinson and Company, NJ, USA).

A WHO-endorsed line-probe assay (LPA) for determining rifampicin and isoniazid resistance, GenoType MTBDR*plus* v2.0 (Bruker-Hain Diagnostics, Germany),^4^^,^^5^ was performed post-culture (median time to positivity 4 days, range 3–7 days). Species identification was performed with the GenoType MTBC assay (Bruker-Hain Diagnostics).

Phenotypic drug susceptibility testing (pDST) was performed using the BD BACTEC MGIT 960 culture system according to the manufacturer’s instructions. The *Mycobacterium tuberculosis* isolates were tested for susceptibility to first-line and second-line anti-tuberculous drugs at WHO-defined critical concentrations: rifampicin (1.0 mg/L), isoniazid (0.1 mg/L), ethambutol (5 mg/L), pyrazinamide (100 mg/L), moxifloxacin (0.25 mg/L), amikacin (1.0 mg/L), linezolid (1.0 mg/L) and clofazimine (1.0 mg/L).^3^ Additional testing to determine rifampicin MICs for each isolate was performed using a broth microdilution method, the Sensititre MYCOTB MIC Plate (TREK Diagnostic Systems, Cleveland, OH, USA) according to the manufacturer’s instructions. Identification of the rifampicin resistance mutation was performed using *rpoB* gene Sanger sequencing.^6^ Epidemiological typing was performed in house using a 24 locus MIRU-VNTR typing kit (GenoScreen, Lille, France).^7^ Phylogenetic lineages were assigned to each isolate using the MIRU-VNTR*plus* online tool.^8^^,^^9^ There were a further six *M. tuberculosis* isolates from the IMRL database, dating from 2010 to the present investigation, with an indistinguishable MIRU-VNTR genotype. WGS was performed on the nine *M. tuberculosis* isolates using an Illumina high output MiniSeq kit (Illumina^®^, San Diego, CA, USA) according to the manufacturer’s instructions. SNP-based analysis was performed using the MTBseq v1.0.4 pipeline.^10^

### Ethics statement

This outbreak was investigated by the local Departments of Public Health under statutory legislation and did not require ethics approval. Legal duties, organizational policies and good practices were observed in data handling and data processing for the study which was conducted by the authors to inform the statutory function of the Health Services Executive in Ireland to improve, promote and protect the health and welfare of the public (Section 7, Health Act 2004), in line with the General Data Protection Regulations and their application in Ireland. This report was approved, prior to submission, by the Data Protection Officer at St James’s Hospital.

### Data availability

Raw sequence reads of all nine sequenced *M. tuberculosis* genomes in this study were submitted to the European Nucleotide Archive database under project accession number PRJEB43194.

## Results

The laboratory data for the three *M. tuberculosis* isolates are shown in Table 1.

**Table 1.** Comparison of genotypic and phenotypic laboratory test results for *M. tuberculosis* isolates harbouring the A451V mutation

Sample no.	Sample type	Xpert^®^ MTB/ RIF Ultra^a^, SMB probe with Tm shift	GenoType MTBDR*plus*v2.0^b^		Rifampicin pDST		*rpoB* mutation^e^
			*rpoB* WT	*rpoB* MUT	BACTEC MGIT liquid culture^c^	broth microdilution MIC (mg/L)^d^	
1	sputum	rpoB4A	WT1-8 present	not detected	S	<0.12	A451V (A532V)
2	sputum	rpoB2, rpoB4A	WT8 absent	not detected	S	<0.12	A451V (A532V)
3	sputum	rpoB2, rpoB4A	WT8 absent	not detected	S	0.25	A451V (A532V)

MUT, mutation/mutant; SMB, sloppy molecular beacon; Tm, melting temperature; S, susceptible .

a Xpert^®^ MTB/RIF Ultra v2 assay used for sample 1; Ultra v3 assay used for samples 2 and 3.

b Assay performed on *M. tuberculosis* recovered from each sample.

c pDST performed using the BD BACTEC MGIT 960 culture system and tested at WHO-defined critical concentration of 1 mg/L rifampicin.

d MIC determined by broth microdilution using TREK^®^ Sensititre MYCOTB MIC Plate.

e *rpoB* mutation identified using Sanger sequencing, confirmed with WGS analysis.

Xpert^®^ MTB/RIF Ultra detected *M. tuberculosis* complex DNA and a rifampicin resistance-associated mutation in the three respiratory samples. Sample 1 displayed slightly different Xpert^®^ results to Samples 2 and 3. The raw data from this test showed that probe rpoB4A was detected in all samples, with a second probe (rpoB2) observed in samples 2 and 3 (both WT and mutation melt curves detected). GenoType MTBDR*plus* LPA results were similar for samples 2 and 3 where rifampicin resistance was inferred from the banding pattern obtained due to lack of signal to one *rpoB* WT probe (WT8 probe not developed; no mutation probe developed).^5^ However, there was no indication of rifampicin resistance from the banding pattern obtained for sample 1 (all WT probes developed, no mutation probe developed). All three samples were confirmed as *M. tuberculosis* and pDST results showed susceptibility to all first- and second-line anti-TB drugs when tested at WHO-defined critical concentrations.^3^ Additional tests showed rifampicin susceptible MICs (shown in Table 1) for each isolate using a broth microdilution method. Sanger sequencing revealed an A451V (codon 532) *rpoB* gene mutation in all three isolates and was confirmed with WGS analysis.

**Figure 1.**
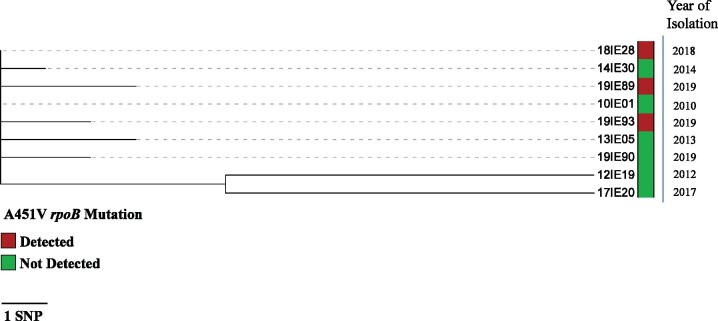
SNP-based analysis performed using the MTBseq v1.0.4 pipeline with minimum stringency threshold confirmed the frequency of the mutant allele at 58% in sample 1 with 88× coverage depth. The frequency of the mutation in samples 2 and 3 was 100% in all instances with coverage in that region 172× and 85×, respectively. A maximum likelihood tree was constructed with the RAxML GUI v2.0.0. The analysis parameters selected were ML + rapid bootstrap with 1000 reps and compute branch lengths selected. The resulting output file was visualized on the interactive tree of life website.

MIRU-VNTR typing showed the three *M. tuberculosis* isolates were indistinguishable and belonged to Euro-American lineage 4 (MtbC 15-9 code: 3199-15). SNP based WGS analysis using the MTBseq pipeline showed that these three isolates’ genomes differed from each other by ≤5 SNPs (Figure 1), which indicated a high likelihood of recent transmission events involving these cases due to the low mutation rate (estimated at 0.5 SNPs per genome per year) of *M. tuberculosis*.^10^^,^^11^ An analysis of all nine sequenced *M. tuberculosis* isolates showed that 16 SNPs separated the most distant of the isolates in this cohort while the majority (7/9), including the isolates from the three index cases, were no more than 6 SNPs apart from each other. Despite extensive Public Health investigations and review of each case and their contacts, no epidemiological links were identified. Two of the three TB cases with the A451V mutation lived in the same town (population 30 000), while the third case lived in the same province as these cases.

## Discussion

WGS has recently become the gold standard for detecting TB transmission and identifying TB outbreaks.^12^ But in order to effectively translate WGS data, real-time epidemiological analysis is required. While this A451V mutation appears to be rare, no epidemiological links were identified between the three Irish-born adult cases harbouring this mutation. Furthermore, no epidemiological links could be established between the seven cases that were ≤6 SNPs apart.

WHO recommends Xpert^®^ MTB/RIF Ultra and GenoType MTBDR*plus* v2.0 as initial tests to detect drug resistance prior to the initiation of appropriate therapeutic regimens.^1^^,^^4^ While these tests are rapid and highly sensitive, they are not considered highly specific for the diagnosis of rifampicin resistance in *M. tuberculosis,* so pDST remains the gold standard.^3^ However, phenotypic methods are time-consuming and are not without limitations. Therefore, WHO now recommends sequencing the entire *rpoB* gene to identify rifampicin resistance-associated mutations.^3^

The difference in Xpert^®^ results between sample 1 and samples 2 and 3 was most likely due to the difference between Ultra v2 and Ultra v3 assays. The rpoB4 probe detected in all three samples covers codons 525–536, which would include the A451V mutation (codon 532). The presence of the second probe, rpoB2, which covers codons 512–525 in samples 2 and 3 suggests a mixed population that was not borne out by sequencing data.^2^ Sanger sequencing and WGS were re-analysed, but no further variants (silent or otherwise) could be found to explain a melting curve change in this region. Neither could the one mutation account for both melt curve changes since their regions do not overlap. Each of the tests was performed using a kit with a different lot number. Also, the WT probe (WT8) that was absent on the LPA covers codons 531–533, which again correlates with A451V and not any other variant.

The A451V mutation being associated with an Xpert^®^ MTB/RIF Ultra report of rifampicin resistance in a clinical sample has not been reported previously. There is one report of an *M. tuberculosis* isolate with an A451V mutation that was phenotypically susceptible to rifampicin, but its clinical relevance was not assessed.^13^ In previous reports where this mutation has been found in rifampicin-resistant strains there was an accompanying high-confidence *rpoB* gene mutation.^14^ Not all *rpoB* mutations have the same effect on rifampicin susceptibility and some have been described as ‘disputed mutations’.^15^ Recently, WHO has revised the rifampicin critical concentration for pDST using the BACTEC MGIT 960 system to capture these ‘disputed mutations’. The rifampicin critical concentration was lowered from 1.0 mg/L to 0.5 mg/L.^16^

In recognizing the potential for discordance between identified *rpoB* gene mutations and phenotypic rifampicin susceptibility results a list of confidence-graded mutations associated with rifampicin resistance has been proposed; this does not include A451V.^17^^,^^18^ A publicly available list of mutations that do not confer phenotypic rifampicin resistance in *M. tuberculosis* is required and would be helpful to guide therapeutic decision-making.

The fact that novel, disputed or unknown variants could alter the melting curves of the Xpert^®^ assay indicates that users should be aware of the possibility of false detection of rifampicin resistance, even though this is most likely a rare occurrence.^19^ In high TB burden countries, it may be appropriate to treat with an MDR regimen in cases where the Xpert^®^ indicates rifampicin resistance. However, in countries where the TB burden is relatively low, the impact of false positive rifampicin resistance is more prominent and waiting for a confirmatory pDST result might be preferable. The significance of disputed or novel mutations is still debatable as there is little knowledge of the consequences of infection with *M. tuberculosis* strains harbouring them.^20^ Furthermore, the frequency of these mutations is unknown as they are likely missed when only pDST is performed.
